# S100b Counteracts Neurodegeneration of Rat Cholinergic Neurons in Brain Slices after Oxygen-Glucose Deprivation

**DOI:** 10.1155/2010/106123

**Published:** 2010-05-24

**Authors:** Daniela Serbinek, Celine Ullrich, Michael Pirchl, Tanja Hochstrasser, Rainald Schmidt-Kastner, Christian Humpel

**Affiliations:** ^1^Laboratory of Psychiatry and Exp. Alzheimer's Research, Department of Psychiatry and Psychotherapy, Innsbruck Medical University, Anichstra*β*e 35, 6020 Innsbruck, Austria; ^2^C. E. Schmidt College of Biomedical Science, Florida Atlantic University (FAU), Boca Raton, FL 33431, USA

## Abstract

Alzheimer's disease is a severe chronic neurodegenerative disorder characterized by beta-amyloid plaques, tau pathology, cerebrovascular damage, inflammation, reactive gliosis, and cell death of cholinergic neurons. The aim of the present study is to test whether the glia-derived molecule S100b can counteract neurodegeneration of cholinergic neurons after oxygen-glucose deprivation (OGD) in organotypic brain slices of basal nucleus of Meynert. Our data showed that 3 days of OGD induced a marked decrease of cholinergic neurons (60% of control), which could be counteracted by 50 *μ*g/mL recombinant S100b. The effect was dose and time dependent. Application of nerve growth factor or fibroblast growth factor-2 was less protective. C-fos-like immunoreactivity was enhanced 3 hours after OGD indicating metabolic stress. We conclude that S100b is a potent neuroprotective factor for cholinergic neurons during ischemic events.

## 1. Introduction

Alzheimer's disease (AD) is characterized by beta-amyloid plaque depositions, tau pathology, inflammation, cerebrovascular damage, and cell death of cholinergic neurons. The lack of cortical acetylcholine directly correlates with cognitive impairment. Loss of function and degeneration of cholinergic neurons in the basal forebrain has been a central theme in AD research, leading to cholinergic neuroprotection strategies for therapy [1]. Loss of trophic support by target neurons in the cortex has been considered as one mechanism for cholinergic degeneration [1]. Furthermore, cholinergic neurons show pathological changes such as accumulation of neurofibrillary tangles and axonal abnormalities early during aging and AD [2–4]. Changes of the basal forebrain are already found in the presymptomatic phase of AD using neuroimaging [5, 6]. Exogenous trophic factors can support cholinergic neurons in vitro and in vivo and replacement strategies have been under consideration for treating AD. Nerve growth factor (NGF) is so far the most potent trophic factor to protect cholinergic neurons against neurodegeneration [7]. However, other trophic molecules are also of considerable interest, such as, for example, S100b.

S100b is a calcium-binding protein predominantly produced in astrocytes [8], but also oligodendrocytes have been shown to express the protein [9]. S100b is released from glial cells and reaches surrounding neurons on which it exerts trophic functions under physiological conditions [8, 10, 11]. Neuroprotection against glutamate toxicity has been shown in neuronal cell culture models [12]. S100b also activates microglial cells and stimulates iNOS or IL1*β* via the NF-kappaB pathway [13, 14]. RAGE (receptor for advanced glycosylation end products.

(AGE)) has also been linked to cellular activation by S100b [15]. S100b has been also linked to AD, because astrocytes are strongly activated in AD and in the brain and tissue levels of S100b are increased [16]. It was generally thought that the astroglial response serves protective functions, including the increased production of trophic factors and uptake of beta-amyloid peptides, but negative aspects of astrocytic activation have also been uncovered [17]. It was postulated that excessive production of S100b could cause damage to neurons in the brain of AD patients [16, 18]. In fact, overexpression of human S100b exacerbated brain damage after ischemia-hypoxia [19, 20], and arundic acid ameliorated ischemic damage by preventing astrocytic overproduction of S100b [21]. Taken together, at low (nanomolar) concentrations S100b exerts trophic functions on neurons, whereas at high (micromolar) concentrations the protein stimulates cytokine production which in turn may trigger apoptosis [10]. 

It has been suggested that chronic neurovascular damage and moderate ischemia-hypoxia (silent strokes) may play a role in the progression of AD [22]. Moderate ischemia and hypoxia can stimulate astrocytes [23]. In the brain afflicted by AD, loss of trophic support coexists with hypoxic conditions stimulating glial cells, resulting in complex interactions that may involve S100b. Ischemic damage of neurons provokes reactions of astrocytes whereby immunohistochemical labeling for S100b is detected in reactive astrocytes and in damaged neurons [24]. 

The aim of the present study is to explore the role of S100b on cholinergic neurons in our well established organotypic brain slice culture model in which cholinergic basal nucle**us** of Meynert (nBM) neurons are provided with NGF for two weeks. Slices incubated without NGF display loss of choline acetyltransferase (ChAT) labeling after two weeks [25] which is an indicator of neurodegeneration. Oxygen-glucose deprivation (OGD) of slice cultures has become an established model for “ischemia-like” conditions [26–28]. In this report, we show that prolonged OGD induces loss of ChAT signals of 2-week old nBM neurons and that S100b can protect against the detrimental effects of OGD. No evidence for a negative effect of S100b itself was detected. The present study suggests that S100b plays a protective role for cholinergic neurons undergoing ischemia-hypoxia stress.

## 2. Materials and Methods

### 2.1. Organotypic Brain Slices

Cholinergic neurons in organotypic brain slices were cultured as described by us in detail [25, 29]. Briefly, the basal nucleus of Meynert of postnatal day 8 (P8) rats was dissected under aseptic conditions, 400 *μ*m slices were cut with a tissue chopper (McIlwain, USA), and the slices were placed on 30 mm Millicell-CM 0.4 *μ*m pore membrane culture plate inserts (7-8 slices per membrane). Slices were cultured in 6-well plates at 37°C and 5% CO_2_ with 1.2 mL/well of slice medium (50% MEM/HEPES (Gibco), 25% heat-inactivated horse serum (Gibco/Lifetech, Austria), 25% Hanks' solution (Gibco), 2 mM NaHCO_3_ (Merck, Austria), 6.5 mg/mL glucose (Merck), and 2 mM glutamine (Merck), pH 7.2) including 10 ng/mL nerve growth factor (NGF) for 2 weeks. It is well-established that the 400 *μ*m brain slices become thinner during the 2 weeks of incubation and have a thickness of approximately 100 *μ*m after 2 weeks. Slices which did not flatten were removed from the experiments. For the experiment the 2-week old slices were cultured for 3 days without NGF and then transferred to different media: (1) medium with normal 33.2 mM glucose or (2) medium with low (6.15 mM) glucose. Recombinant S100b from bovine brain (Calbiochem) was added to the medium at concentrations from 0.1–50 *μ*g/mL. Alternatively, 10 ng/mL NGF or 2 ng/mL fibroblast growth factor-2 (FGF-2) was added. It has to be noted that slices could not be cultured in medium without any glucose, because then slices become shrunken and cannot be evaluated.

### 2.2. Oxygen-Glucose Deprivation (OGD)

Slices in the wells (with low glucose) were transferred to a Modular Incubator Hypoxia Chamber (MIC-101, Billups-Rothenberg, Inc., Del Mar, CA, USA) connected to a flow meter. The chamber was sealed and a mixture of 95% N_2_/5% CO_2_ was flushed at a flow rate of 25 L/min for 5 minutes. The in- and out-ports of the chamber were closed, and the closed air tight system was placed at 37°C in an incubator for 1, 2, or 3 days. Measurements by the manufacturer indicate that the pO_2_ reached a nadir of 35 mm Hg after 6 hr. At the end of the experiment the outlet port was slowly opened, the chamber opened, and the culture wells taken out and slices fixed for 3 hours with 4% paraformaldehyde at 4°C. Control slices were kept at normal 5% CO_2_/air conditions in the incubator at 37°C.

### 2.3. Immunohistochemistry

Immunohistochemistry using the avidin-biotin technique was performed to detect cholinergic neurons as described [30, 31]. All incubations for immunohistochemistry were performed free-floating at 4°C for 2 days including 0.1% Triton, which allows good penetration of the antibody into the slices from both sides. Fixed slices were washed for 30 minutes with 0.1% Triton/PBS (T-PBS) at room temperature and pretreated for 20 minutes with 5% methanol/1% H_2_O_2_/PBS. Then the slices were rinsed three times for 10 minutes with PBS, blocked with 20% horse serum/0.2% BSA/T-PBS, and then incubated with the primary antibody against ChAT (1 : 750, Millipore) or c-fos (1 : 1000, Santa Cruz) in 0.2% BSA/T-PBS for 2 days at 4°C. Slices were washed and incubated with secondary biotinylated anti-goat (ChAT) or anti-rabbit (c-fos) antibody (1 : 200, Vector Laboratories), for 1 hour at room temperature. After rinsing three times in PBS, slices were incubated in avidin-biotin complex solution (ABC; Elite Standard PK 6100, Vector Laboratories) for 1 hr, then washed three times in 50 mM Tris-buffered saline (TBS), and the signal was detected using 0.5 mg/mL 3,3′diaminobenzidine (DAB) in TBS with 0.003% H_2_O_2_ as substrate. Slices were then rinsed in PBS and mounted on gelatine-coated glass slides.

### 2.4. Quantitative Analysis and Statistics

All neuronal counts were based on individual sections and show total number of neurons per slices. The number of microscopically detectable immunoreactive ChAT^+^ neurons was counted in the whole slice visualized under a 20× objective by an investigator blinded to the treatment code. Multistatistical analysis was obtained by one-way ANOVA, followed by a subsequent Fisher PLSD posthoc test by comparing controls against the respective treatments, where *P* < .05 represents statistical significance.

## 3. Results

**Figure 1:**
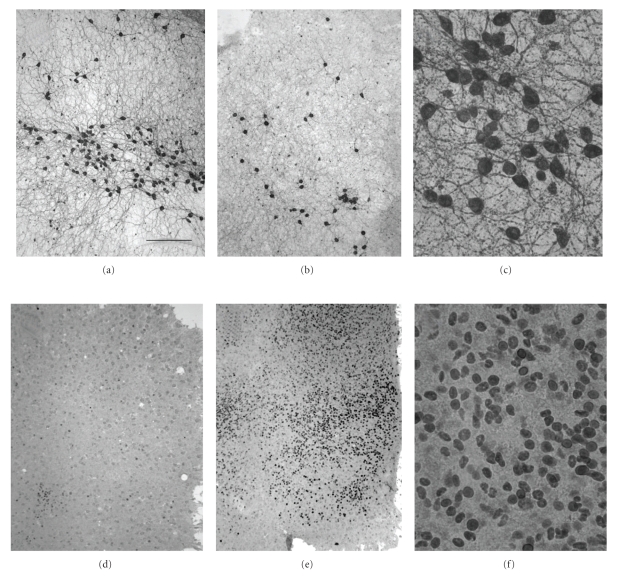
Immunohistochemistry for cholinergic choline acetyltransferase (ChAT) positive neurons (a)–(c) and c-fos immunoreactive nuclei (d)–(f) in organotypic brain slices of the basal nucleus of Meynert. Slices show control stainings before ((a), (c), (d)), or 3 days (ChAT^+^, (b)) or 3 hours (c-fos+, (e) and (f)) after oxygen-glucose deprivation. Scale bar in *A* = 300 *μ*m ((a), (b), (d), and (e)) and 75 *μ*m ((c) and (f)).

When brain slices were cultured for 2 weeks in medium with normal glucose levels containing 10 ng/mL NGF, approximately 140 cholinergic nBM neurons/slice were detectable (Table 1; Figures 1(a) and 1(c)). This number did not change when slices were incubated without NGF for further 3 days (Table 1). Reduction of glucose to 6 mM (glucose deprivation) did not significantly change the number of cholinergic neurons after 1-2-3 days of incubation (Table 1). Slices exposed to hypoxia at normal (high) glucose did not show a reduced number of ChAT^+^ neurons after 1-2-3 days of incubation (Table 1). However, when slices were incubated in low glucose and hypoxia (oxygen-glucose deprivation, OGD), the number of ChAT^+^ neurons significantly decreased to about 70 neurons/slice after 3 days but not after 1 or 2 days of incubation (Table 1; Figure 1(b)).

In order to test whether S100b counteracts degeneration of cholinergic neurons caused by OGD for 3 days, slices were incubated with 50 *μ*g/mL of S100b during the exposure. S100b counteracted the decrease of ChAT^+^ neurons after 3 days of OGD (Table 1). By comparison NGF also counteracted loss of ChAT^+^ neurons but was less potent (Table 1). FGF-2 only slightly counteracted the OGD effect (Table 1). Incubation of slices with S100b alone did not exert any toxic effect on ChAT expression in cholinergic neurons (Table 1).

In order to verify stressful effects of OGD on neurons, slices were stained for c-fos immunoreactivity at 3 and 24  hours of OGD (Figures 1(d)–1(f)). In controls without OGD, immunohistochemistry showed very few c-fos positive nuclei (Figure 1(d)). The number and intensity of c-fos positive nuclei significantly (*P* < .001) increased at 3 hours of OGD (Figure 1(e) and 1(f)) and thereafter returned to control levels at 24 hours of OGD.

## 4. Discussion

In the present study we show that cholinergic neurons of the basal nucleus of Meynert undergo neurodegeneration when exposed during 3 days of oxygen-glucose deprivation in a slice culture model. This neurodegeneration could be counteracted by recombinant S100b which is an endogenous glial protein. 

### 4.1. Cholinergic Neurons in Brain Slices

The cholinergic neurons of the basal forebrain are a functionally homogeneous population, localized in the medial septum/diagonal band of Broca and basal nucleus of Meynert (nBM), provide the major cholinergic innervation to the hippocampus/amygdala and neocortex, respectively, and play a crucial role in cognition and memory. In neurodegenerative disorders such as AD, these neurons are dysfunctional and can further degenerate in the progression of the disease [32]. To study the mechanisms involved in neurodegenerative processes, as well as neuroprotective strategies, the organotypic brain slice model has been established [25, 33–37]. Detection of cholinergic neurons was performed using the immunohistochemical marker for the enzyme ChAT, which is expressed in the cell bodies and nerve fibers. A decreased number of ChAT^+^ neurons directly correlates with neurodegeneration of cholinergic neurons. In our brain slice model cholinergic nBM neurons are axotomized and the number of ChAT^+^ neurons is markedly decreased without exogenous NGF. Application of recombinant NGF counteracts this degeneration, displaying a slight neuroprotective effect at a concentration of >0.1 ng/mL NGF and full activity at >1 ng/mL NGF [37]. Thus, our brain slice model may allow to study the cholinergic phenotype or the degeneration of the majority of cholinergic neurons, which is seen by shrunken neurons and loss of nerve fibers and a decrease of the ChAT^+^ immunoreactivity. In order to test whether S100b exerts toxicity on cholinergic neurons, 2-week slices were exposed to a relatively high dose of 50 *μ*g/mL (2.38 *μ*M) of S100b for 3 days, but S100b did not affect the cholinergic neurons in our slice model. It is unlikely that a toxic effect would have shown with short exposures of 1 or 2 days.

### 4.2. Oxygen-Glucose Deprivation as a Model of Ischemia

The OGD model in slice cultures has been widely used to study “ischemia-like” conditions [26–28]. Most of the studies have used a strong and transient insult in slice cultures of the hippocampus to model the selective vulnerability to ischemia that is observed in vivo after cardiac arrest [26]. Neuronal damage is typically evaluated by dye-uptake and imaging techniques to quantify treatment effects [28]. The present model used a mild and more prolonged OGD impact (i.e., 1–3 d) to simulate the blood flow disturbances in the brain afflicted by vascular changes in AD. The evaluation was focused on the cholinergic neurons in the nBM using a high-resolution approach with neuronal counting. OGD in slice culture eliminates the influence of blood flow, which otherwise is a complex variable in animal models of brain ischemia. The observation that neurons are more vulnerable to ischemia (= combined loss of oxygen and glucose) than to either hypoglycemia or hypoxia alone has been made in multiple experimental conditions and probably applies to the clinical situation as well. One has to recognize that experimental OGD induces strong reductions but not total removal of glucose and/or oxygen. Since the slices are a stable system, the effects of OGD alone are very weak after 3 days. We observed reductions of cholinergic neurons but no widespread cell damage. In the case of glucose deprivation (GD), endogenous stores are not completely exhausted within three days and alternative metabolites may be used. We have data showing that glucose reduction alone for 2 weeks markedly reduced cholinergic neurons. Oxygen deprivation (= hypoxia) is tolerated because of anaerobic glycolysis based on glucose stores which last for three days. Oxygen deprivation longer than 3 days is problematic, because the pH markedly decreases due to lactate production. 

It should be noted that vascular networks persist in the slice culture in the absence of blood flow [35, 38]. The slice model should provide the opportunity to study reactions of vascular cells to ischemia-like conditions in the absence of intraluminal inflammatory cells. This is an important aspect, because endothelial cells can produce potentially damaging factors for cholinergic neurons [36]. In order to show that cellular stress is initiated soon after onset of oxygen-glucose deprivation, we analyzed the prototypic immediate early gene c-fos, which is upregulated within minutes in response to a strong stimulus, including ischemia, and rapidly downregulated in most experimental systems, even if the stimulus persists. For this purpose it was sufficient to show that c-fos was increased at 3 hours.

### 4.3. S100b Protects Cholinergic Neurons

The present finding that S100b protected cholinergic neurons in the slice culture model against OGD supplements previous reports of a protection of hippocampal neurons from glucose deprivation [39]. At low concentrations, S100b exerts multiple positive effects on neurons, including control of protein phosphorylation, regulation of energy metabolism and function of the cytoskeleton [8, 10]. Autocrine effects on astrocytes have also been described [10]. The role of S100b stimulation of the RAGE receptor in protection remains to be understood [10]. While there are reports of damaging effects of S100b in high concentrations [10], our experimental data do not suggest a negative influence on cholinergic neurons in vitro, at least for 3 days. In our study we tested different concentrations of 0.1–50 *μ*g/mL but only the highest dose of 50 *μ*g/mL (=2.38 *μ*M) provided protection against oxygen-glucose deprivation. It has been reported that oxygen-serum-glucose deprivation induces release of S100b in the nanomolar concentration from astrocytes after 24  hours [11]. In our slice experiments 0.1 *μ*g/mL S100b did not provide protection. Indeed, we assume that in our model of oxygen-glucose deprivation, the intrinsic astrocytes do release S100b but this may be insufficient to provide rescue of cholinergic neurons in this insult. A 100-fold higher dose was required to provide protection in our model system with oxygen-glucose deprivation but it is difficult to compare concentrations obtained in different experimental systems. Our organotypic brain slice cultures have a thickness of 100-150 *μ*m by two weeks after explantation and it seems likely that a higher dose is necessary for diffusion deep into slices, so that concentrations reached at the level of the cell-bodies several tens of microns deep in the slice may be much lower.

### 4.4. An Expanded Role for Astrocytes in Alzheimer's Disease

Glial cells surrounding the basal forebrain cholinergic neurons are known to provide important paracrine trophic support, including NGF, FGF-2, epidermal growth factor, brain-derived neurotrophic factor, and ciliary neurotrophic factor. S100b could be an additional trophic factor supplied by local glial cells to cholinergic neurons, and theoretically, a decline of S100b production could contribute to cholinergic dysfunction. So far, most studies have focussed on the activation of astrocytes in the AD brain which are often related to senile plaques [40]. However, a reduction of glial function could be part of the complex pathophysiology of AD and improving glial function should be protective [17]. It is notable that two of the major risk genes for late-onset AD, that is, APOE and CLU/Clusterin [41], have important functions in astrocytes [42, 43], and gene variants could relate to reduced glial functions. Thus, complex interactions between the primary processes in AD (beta-amyloid plaque depositions, tau pathology), moderate ischemia-hypoxia, and genetic variations of glial reactions can be envisaged. In fact, it has been reported that S100b protects LAN-5 neuroblastoma cells against beta-amyloid-induced neurotoxicity at lower nanomolar doses, while S100b was toxic to LAN-5 cells at micromolar doses [44]. In their study [44] individual cells were exposed directly to S100b, whereas in our model the protein had to diffuse into the slices. In fact, neurons or glial cells in isolation on culture dishes do not have protection from surrounding cells and are exposed to high levels of radical oxygen species. Our slice model may incorporate these endogenous protective mechanisms. One could argue that our model using a tissue explant is closer to the in vivo situation than cell cultures. Another explanation is also that the recombinant S100b is derived from the bovine gene, while we use rat brain slices, and a mismatch in protein sequence and/or altered dimeric/monomeric forms may contribute to a lower functional efficiency on rat brain S100b sensitive receptors.

In conclusion the present study shows that 3 days of oxygen-glucose deprivation induced a marked decrease of cholinergic neurons, which could be counteracted by S100b. We conclude that S100b is a potent neuroprotective factor for cholinergic neurons after ischemic events.

## Figures and Tables

**Table 1:** Cholinergic neurons after glucose-oxygen deprivation and neuroprotection with S100b.

Treatment		ChAT^+^ neurons	p1	p2
Day 0		136 ± 17 (14)	—	
+1d	GD	128 ± 17 (5)	ns	
	OD	131 ± 27 (6)	ns	
	OGD	113 ± 22 (6)	ns	—
	OGD + S100b (50 *μ*g/mL)	121 ± 34 (3)		ns
+2d	GD	130 ± 21 (5)	ns	
	OD	124 ± 21 (8)	ns	
	OGD	110 ± 25 (6)	ns	—
	OGD + S100b (50 *μ*g/mL)	138 ± 25 (6)		ns
+3d	(-)	138 ± 10 (18)	—	
	S100b (50 *μ*g/mL)	139 ± 18 (19)	ns	
	GD	122 ± 16 (13)	ns	
	OD	135 ± 12 (12)	ns	
	OGD	81 ± 8 (28)	∗∗∗	—
	OGD + S100b (50 *μ*g/mL)	130 ± 11 (26)	ns	∗∗∗
	OGD + S100b (1 *μ*g/mL)	89 ± 10 (14)	∗∗	ns
	OGD + S100b (0.1 *μ*g/mL)	92 ± 12 (14)	∗	ns
	OGD + FGF-2 (2 ng/mL)	102 ± 9 (12)	*P* = .06	ns
	OGD + NGF (10 ng/mL)	94 ± 12 (18)	∗	ns

Brain slices of the nBM were cultured for 2 weeks with 10 ng/mL nerve growth factor (NGF), then for 3 days without NGF and then treated under oxygen-glucose deprivation (OGD) with or without S100b or NGF or fibroblast growth factor-2 (FGF-2) for 1–3 days. Values are given as mean ± SEM (*n* in parentheses). Statistical analysis was performed by one-way ANOVA with a subsequent Fisher PLSD posthoc test and compared against the untreated control (p1) or against OGD (p2) (*P < .05; **P < .01; ***P < .001; ns not significant). GD, glucose deprivation; OD, oxygen deprivation; OGD, oxygen and glucose deprivation.

## Abbreviations

AD: Alzheimer's disease
ChAT: Choline acetyltransferase
nBM: Basal nucleus of Meynert
NGF: Nerve growth factor
OGD: Oxygen-glucose deprivation.
